# DNA adenine methylation influences gene expression and biofilm formation in *Streptococcus mutans*

**DOI:** 10.1128/aem.01094-25

**Published:** 2025-09-23

**Authors:** Haowei Zhao, Delphine Dufour, Niki Ghobaei, Laurent Bozec, Céline M. Lévesque

**Affiliations:** 1 Faculty of Dentistry, University of Toronto 70374, Toronto, Ontario, Canada; Indiana University Bloomington, Bloomington, Indiana, USA

**Keywords:** *Streptococcus mutans*, biofilm, adenine methylation, restriction-modification system, extracellular DNA, membrane vesicle

## Abstract

*Streptococcus mutans*, a key oral pathogen, utilizes quorum sensing to regulate biofilm formation—a major virulence factor in the development of dental caries (tooth decay). Our recent research uncovered a complex interplay between the CSP-ComDE quorum sensing pathway and the Type II DpnII restriction-modification (R-M) system in *S. mutans*. The DpnII R-M system methylates adenine at 5′-GATC-3′ sites and cleaves unmethylated DNA, significantly influencing foreign DNA acquisition and gene expression. In this study, we investigated the impact of a ΔRM mutant, which lacks adenine methylation, on biofilm formation. The ΔRM mutant formed fragile biofilms that easily detach from surfaces, with significantly reduced exopolysaccharide content and increased extracellular DNA, which appears to be associated with membrane vesicle production rather than cell lysis. RNA-seq analysis revealed only few differentially expressed genes directly involved in biofilm formation, such as *gtfC*, suggesting that the biofilm defect may result from indirect effects or alternative regulatory mechanisms. Notably, the downregulation of mutanobactin-related genes and upregulation of genes involved in *de novo* purine nucleotide biosynthesis point to novel pathways influenced by DNA methylation. These findings contribute to a deeper understanding of the multifactorial nature of biofilm formation and the role of epigenetic modifications in microbial behavior.

**IMPORTANCE:**

This study highlights the critical role of DNA methylation in regulating biofilm formation and virulence in *Streptococcus mutans*. By examining the interplay between adenine methylation, extracellular DNA (eDNA), membrane vesicles (MVs), and glucan production, we provide new insights into the complex biology of biofilm development. Our findings challenge traditional views by emphasizing the importance of MVs and eDNA in maintaining biofilm integrity. Understanding these epigenetics modifications not only advances our knowledge of microbial regulation but also identifies novel targets for antimicrobial therapy. Since adenine methylation is rare or absent in mammalian cells, targeting this modification presents a promising strategy to disrupt biofilm formation and combat bacterial infections. The insights gained from this study may inform the development of innovative approaches to manage biofilm-associated infections and improve oral health outcomes.

## INTRODUCTION

In bacteria, enzymatic DNA methylation primarily occurs post-replication, catalyzed by DNA methyltransferases that transfer a methyl group from S-adenosylmethionine to specific base positions within the DNA strand (1). Among the three major forms of DNA methylation—N6-methyladenine (m6A), 5-methylcytosine (m5C), and N4-methylcytosine (m4C)— methylation of adenine at the N6 position, particularly within 5′-GATC-3′ motifs, is the most prevalent in bacteria (2, 3). The enzymes responsible for DNA methylation often function alongside restriction enzymes as part of bacterial innate immunity, known as the restriction-modification (R-M) system (4). R-M systems typically include a modification enzyme that methylates the host genome at specific DNA sequences and a corresponding endonuclease that cleaves unmodified DNA (4, 5). Accumulating evidence suggests that bacterial DNA methylation, beyond its role in self-DNA protection, also plays a regulatory role in gene expression, influencing the expression of virulence factors (6). The presence of methylated sites in promoter or regulatory sequences can affect gene expression by regulating the binding of RNA polymerase or transcriptional regulators (7, 8). For instance, in *Pseudomonas syringae*, adenine methylation modulates the expression of genes involved in biofilm formation (9), while in uropathogenic *E. coli*, it affects multiple cellular processes, including growth rate, antimicrobial susceptibility, biofilm development, gene expression, and attachment to mammalian cells (10). In line with these regulatory roles, some DNA methyltransferase genes associated with Type I and Type III R-M systems undergo phase variation, resulting in reversible ON/OFF expression states that can further influence gene expression patterns (11).

Biofilms represent a predominant microbial lifestyle and are a major cause of chronic infectious diseases due to their high tolerance to antimicrobial treatments and immune responses (12, 13). These complex microbial communities are embedded in a self-produced extracellular matrix composed of extracellular DNA (eDNA), proteins, and polysaccharides, all of which are critical for structural integrity and biofilm resilience (14–16). Membrane vesicles (MVs)—nanoscale structures released by bacteria—play an important role in this matrix by serving as delivery vehicles for biomolecules that support biofilm development (17, 18). Although gram-positive bacteria possess a thick peptidoglycan layer, which was once thought to hinder vesicle formation, studies since the 1990 s have shown that they do produce extracellular MVs (19, 20). Given the complexity of biofilm architecture—including the extracellular matrix and MVs—a comprehensive understanding of biofilm components and their interactions is essential for developing targeted therapies to disrupt biofilm formation and effectively treat biofilm-associated infections. In the oral cavity, biofilms that develop on tooth surfaces are commonly referred to as dental plaque biofilm (21–23).

*Streptococcus mutans*, a prevalent inhabitant of the human oral cavity, has long been associated with dental caries (tooth decay) due to its well-characterized cariogenic traits (24). While the etiology of dental caries is now recognized as a polymicrobial and ecologically driven process, *S. mutans* remains a key contributor in many individuals, particularly under conditions that favor its dominance. This species exhibits three major characteristics that enhance its cariogenic potential: the ability to synthesize abundant glucan from sucrose, facilitating bacterial colonization; the capability to transport and ferment a wide range of carbohydrates into organic acids, leading to the acidification of the local environment; and the ability to thrive under environmental stress, especially low pH conditions (25). *S. mutans* participates in dental plaque formation through both sucrose-dependent and sucrose-independent mechanisms, with sucrose-dependent adhesion playing a crucial role in altering plaque ecology and promoting caries development (23, 26). Key factors involved in biofilm formation under sucrose-dependent conditions include glucosyltransferases (GTFs) and glucan-binding proteins (GBPs) (27). In contrast, sucrose-independent adhesion involves surface-associated proteins such as antigen I/II (also known as P1, SpaP, or PAc) and WapA, as well as cell envelope-associated proteins like AtlA and BrpA, which also contribute to *S. mutans* biofilm formation (28). Multiple regulatory systems have also been implicated in biofilm formation, including transcription factors, acetylation modifications, two-component systems, CRISPR-associated proteins, and cell-to-cell communication mechanisms (25, 26, 29). Quorum sensing (QS) is a bacterial cell-to-cell communication process that regulates gene expression in response to population density, playing a central role in biofilm formation (30). In *S. mutans*, the best-studied QS system is the CSP-ComDE, composed of the CSP signaling molecule (a small linear peptide of 18-aa residues) and the ComDE two-component system (31–33). This system not only regulates genetic competence for DNA uptake but also influences the expression of a Type II R-M system, DpnII R-M, which protects bacterial cells from foreign DNA (34, 35). By coordinating these processes, QS ensures that biofilm formation and DNA protection mechanisms are optimally regulated, enhancing bacterial survival and pathogenicity (36, 37).

Building on our understanding of the DpnII R-M system’s role in *S. mutans*, our previous work identified this system as responsible for the DNA adenine methylation (DAM) pattern of its genome (35). This R-M system, which includes the methyltransferases DpnM and DpnA, not only plays a role in DNA acquisition but also impacts bacterial physiology, such as cell auto-aggregation (35). Notably, an earlier study by Banas’s group first reported that a component of this system influences the expression of virulence-associated genes in *S. mutans* (38). However, that study focused on a mutant lacking only the first methylase (corresponding to *dpnM*) and did not assess the full impact of DAM loss as the second methylase (*dpnA*) remained intact. In contrast, our current study employs a ΔRM mutant lacking both methyltransferases, which we have shown results in a near-complete loss of DAM (35). This enables a more comprehensive investigation of the role of DAM in *S. mutans* physiology. In this study, we further investigate the role of DAM in *S. mutans* biofilm formation. We demonstrate that the absence of DAM impairs biofilm formation and leads to alterations in the biofilm matrix, including decreased exopolysaccharides and increased eDNA levels, which are linked with MV production. Transcriptomic analysis revealed the differential expression of a limited number of genes. Notably, the gene *gtfC*, which encodes glucosyltransferase C, a product that catalyzes the synthesis of glucans, was significantly repressed in the ΔRM mutant. Additionally, our findings highlight the downregulation of mutanobactin-related genes, which are involved in bacterial survival under oxidative stress (39, 40). Furthermore, the upregulation of purine nucleotide biosynthesis genes suggests an unexpected link between purine metabolism and biofilm formation. Altogether, these findings suggest that DAM plays a crucial role in regulating gene expression, making transcription responsive to DNA methylation. This highlights the broader impact of DAM on the genetic regulatory networks involved in biofilm formation.

## RESULTS

### Deficiency in DAM affects *S. mutans* biofilm adhesion

**Fig 1:**
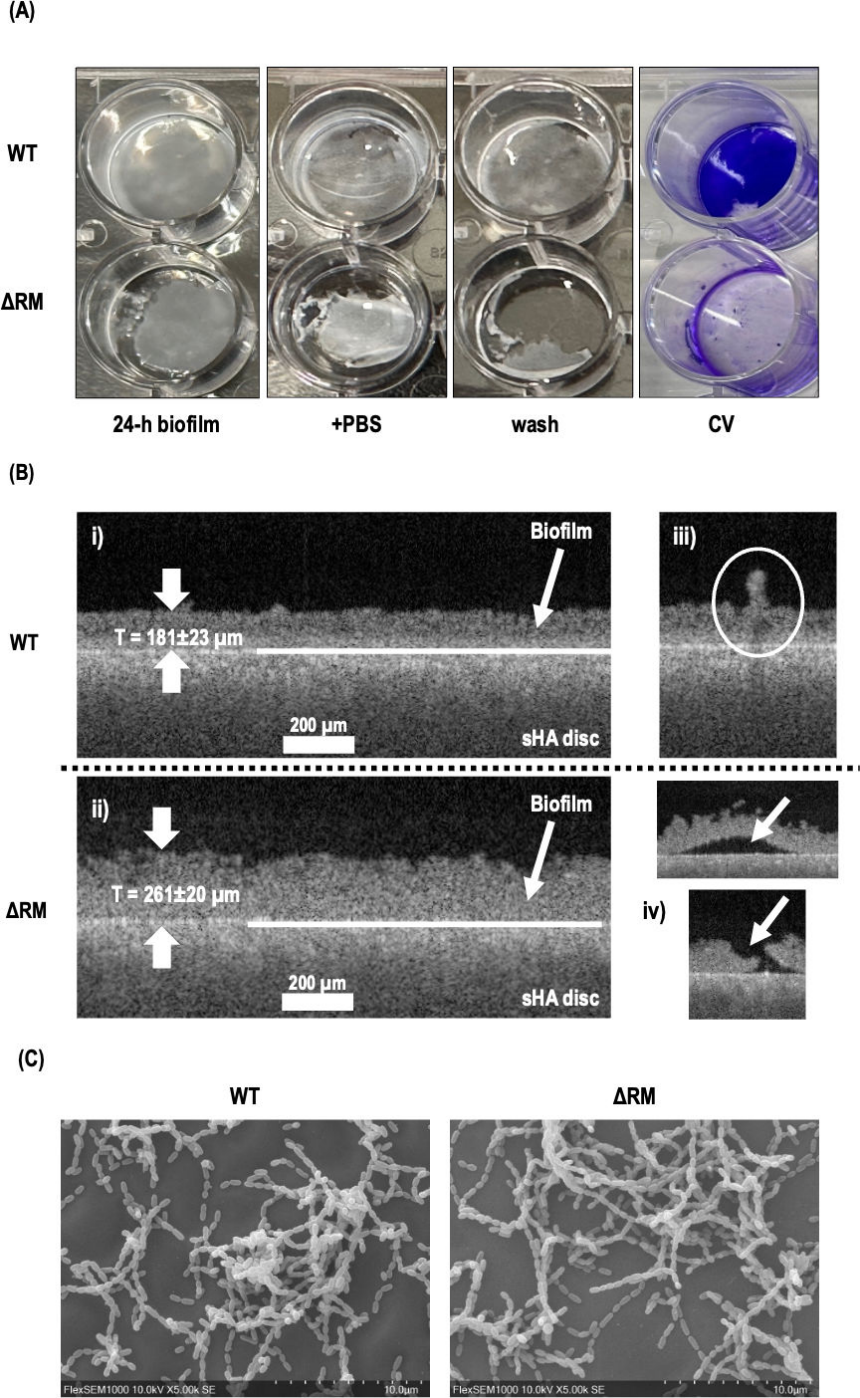
Effect of ΔRM on *S. mutans* biofilms. (**A**) Static biofilms (24-h-old) were developed in BHI-sucrose using microtiter plates. Biofilms were washed with PBS (pH 7.2) and stained with crystal violet (CV). Representative images are shown from triplicate samples. (**B**) Representative 2D OCT images from *S. mutans* WT and ΔRM mutant biofilms attached to sHA disks. A thick horizontal white line in each image delineates the interface between the disk and the biofilm. Biofilm thickness was quantified as the material above this line using ImageJ (*P* < 0.05; *N* = 7). (**C**) Scanning electron micrographs of WT and ΔRM mutant 6-h-old biofilms (magnification, ×5,000).

Our results showed that the *S. mutans* ΔRM mutant, which lacks DAM, exhibited significantly weaker attachment to the substratum compared to the *S. mutans* wild-type (WT) strain. Biofilms formed by the ΔRM mutant were notably more fragile and easily dislodged during the washing step (Fig. 1A), unlike the robust biofilms produced by the WT strain (Fig. 1A; CV staining). To further examine the biofilm architecture, we used optical coherence tomography (OCT) to capture cross-sectional images (Fig. 1B). Two-dimensional (2D) OCT images were obtained from biofilms attached to saliva-coated hydroxyapatite (sHA) disks. Both WT (panel i) and ΔRM mutant (panel ii) appeared to possess a smooth biofilm surface, although some “mushroom-like” structures could be seen for the WT biofilms (panel iii). While the ΔRM mutant exhibited increased thickness compared to the WT strain, its biofilms appeared to have a more open and loosely packed architecture, suggesting reduced cohesion and structural integrity. Notably, panel iv shows the ΔRM mutant biofilm lifting off the sHA surface, further illustrating its compromised integrity and reduced adhesion. These revealed disorganized and fragile biofilms in the ΔRM mutant, while the WT biofilms displayed more cohesive and structured formation. Despite using scanning electron microscopy to visualize the biofilm surface morphology, no significant differences were observed between the WT and ΔRM mutant biofilms (Fig. 1C), suggesting that the primary impact of DAM deficiency is on biofilm adhesion rather than surface structure.

To ensure that the observed phenotype was not strain-dependent, we tested strain UA140, which also encodes the DpnII R-M system (35). The UA140 ΔRM mutant displayed biofilm adhesion properties similar to those observed in the UA159 ΔRM mutant (Fig. S1 in the supplemental material), confirming that the effects of DAM deficiency are most likely consistent across different *S. mutans* strains. Importantly, complementation with DpnM and DpnA methyltransferases restored the WT phenotype in both the UA159 and UA140 ΔRM mutants, resulting in biofilms that were comparable in robustness and adhesion to those of the WT strains (Fig. S1 in the supplemental material). This indicates that the methylation activity from the DpnII R-M system is crucial for maintaining normal biofilm adhesion properties in *S. mutans*.

### DAM does not affect cell surface hydrophobicity or zeta potential

**Fig 2:**
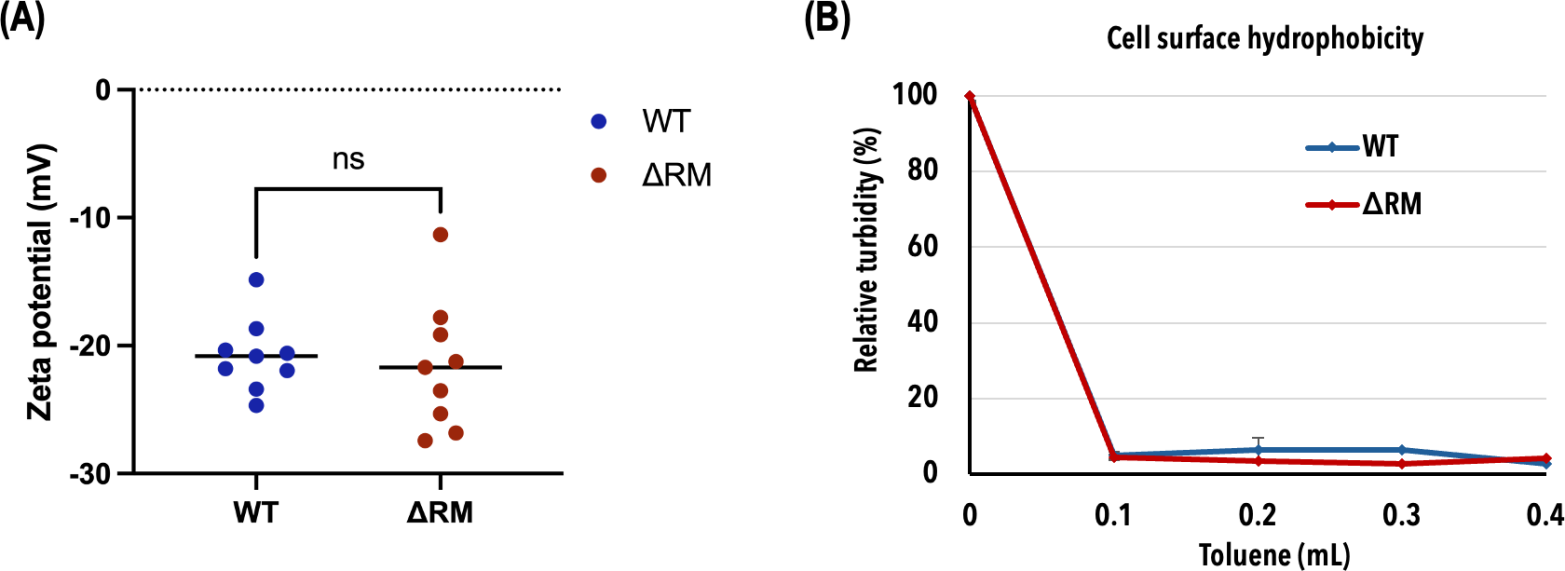
Cell surface properties. (**A**) Surface zeta potential measurements of WT and ΔRM cells in 10 mM KCl, showing no significant (ns) difference (*P* > 0.05; *N* = 3). (**B**) Affinity of WT and ΔRM cells for toluene phase. Fixed-volume aqueous bacterial suspensions were mixed with varying volumes of toluene, and results are expressed as the percentage of turbidity of the aqueous suspension relative to the initial turbidity (*P* > 0.05; *N* = 3).

Given the defective adherence of the ΔRM mutant biofilms to sHA disks and polystyrene microtiter plates, we explored the influence of DAM on various surface properties of *S. mutans* cells. Measurements of zeta potential revealed that both WT and ΔRM cells had comparable zeta potential values, indicating that DAM does not alter the electrostatic properties of the cell surface (Fig. 2A). Furthermore, toluene affinity assays showed that both WT and ΔRM cells exhibited similarly high levels of surface hydrophobicity, suggesting that the absence of DAM does not impact the hydrophobic nature of the cell surface (Fig. 2B). Consequently, we propose that the observed biofilm phenotype may be due to modifications in the biofilm matrix composition or structure rather than changes in cell surface properties.

### Absence of DAM affects the levels of exopolysaccharides and eDNA in the biofilm matrix

**Fig 3:**
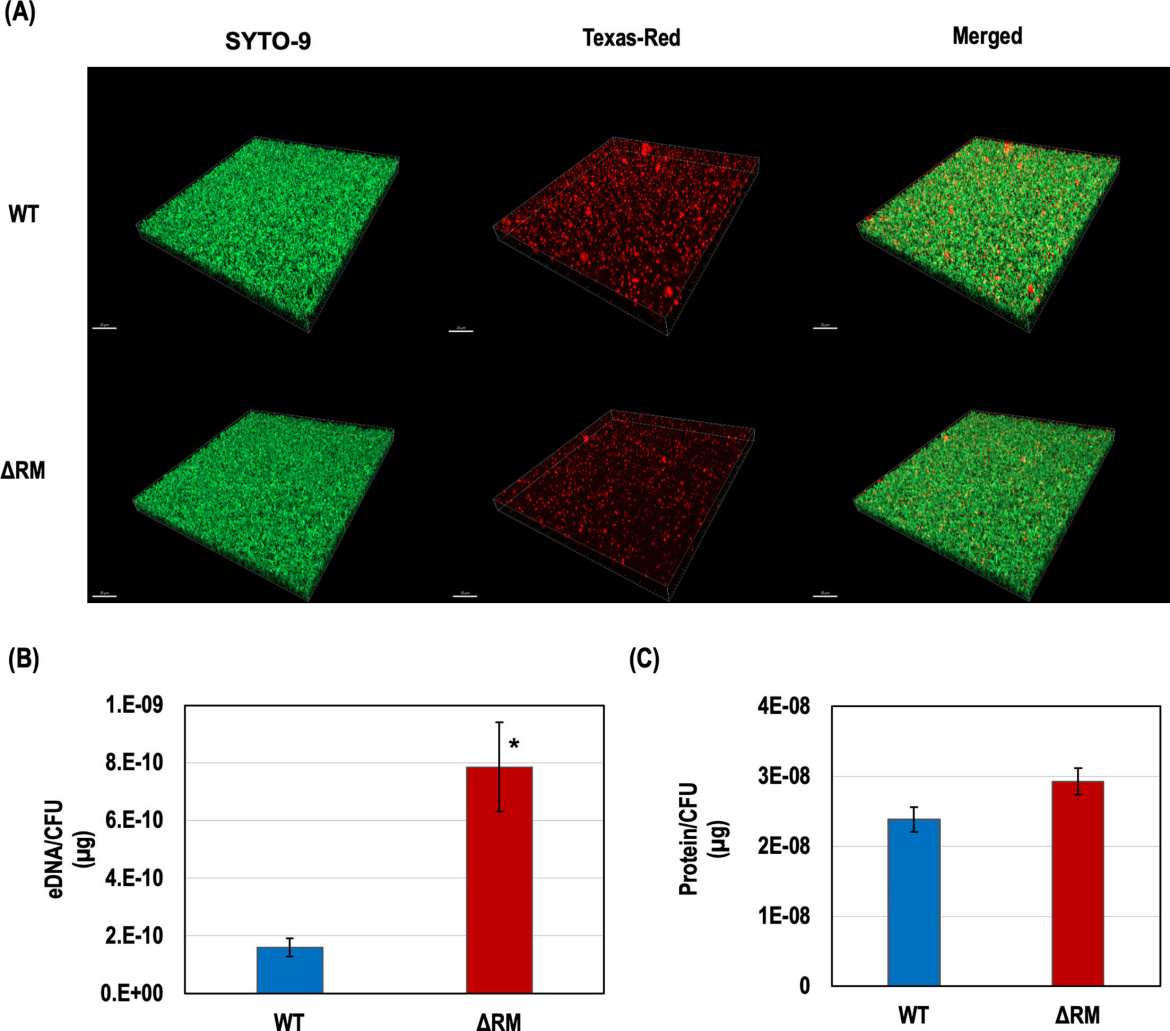
Analysis of biofilm matrix. (**A**) CLSM 3D images of 24 h biofilms developed by the WT strain and ΔRM mutant, with double labeling: SYTO-9 (green) for bacterial cells and Texas-Red-labeled dextran (red) for exopolysaccharides (magnification, ×40; *N* = 10). (**B**) Quantification of eDNA using SYTO-9, which stains both double-stranded and single-stranded DNA (*N* = 3). (**C**) Quantification of protein levels by Bradford assay. * denotes a statistically significant difference compared to WT (*P* < 0.05; *N* = 3).

Quantification of exopolysaccharides in the biofilm matrix revealed that the biovolume of this specific component in the ΔRM biofilm was significantly lower than in the WT strain (*P* = 0.0002), with values of 12.33 ± 1.03 µm^3^/µm^2^ for WT and 9.80 ± 0.80 µm^3^/µm^2^ for the ΔRM mutant. These measurements, based on Texas Red-labeled glucan, reflect the volume of exopolysaccharides alone and not the entire biofilm structure. Confocal laser scanning microscopy (CLSM) 3D images further illustrated this reduction in exopolysaccharide biovolume within the ΔRM biofilm matrix (Fig. 3A). In contrast, earlier observations (Fig. 1B) showed that the ΔRM mutant forms a thicker overall biofilm, which includes bacterial cells and all matrix components. This suggests that while the ΔRM biofilm is structurally thicker, it is composed differently, with reduced exopolysaccharide content. Analysis of eDNA levels showed a significant increase in the ΔRM biofilm matrix, being approximately five times greater than in the WT biofilm matrix (Fig. 3B). Additionally, the protein content in the biofilm matrix was assessed using the Bradford assay, which indicated no significant difference between ΔRM and WT strains (Fig. 3C). These results suggest that the absence of DAM alters the structural components of the biofilm matrix, specifically by reducing exopolysaccharide synthesis and elevating eDNA levels. Consequently, these changes may compromise the overall integrity and stability of the biofilm.

### Enhanced eDNA levels in ΔRM biofilms linked to MV production

**Fig 4:**
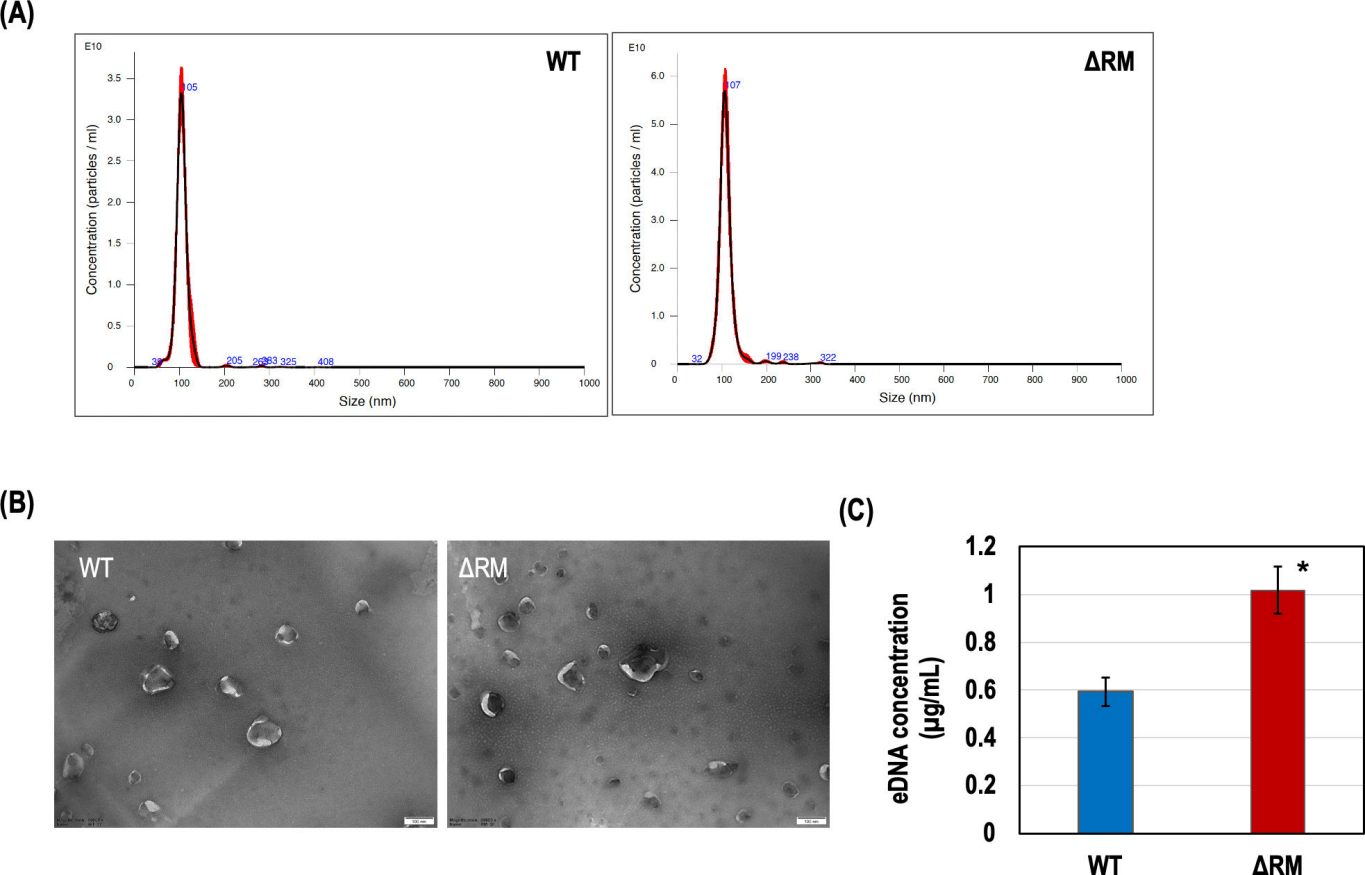
*S. mutans* MVs in biofilms. (**A**) Averaged vesicle concentration for WT and ΔRM biofilms. Samples were diluted 1 in 500, resulting in original concentrations of 9.2 ± 0.4 (×10^11^) particles/mL for WT and 15.8 ± 1.0 (×10^11^) particles/mL for ΔRM. (**B**) TEM micrographs of MVs extracted from WT and ΔRM biofilms (magnification ´60,000). (**C**) Concentration of eDNA in MV suspensions, showing significant differences between WT and ΔRM biofilms. * denotes a statistically significant difference compared to WT (*P* < 0.05; *N* = 3).

To investigate the source of increased eDNA in the ΔRM biofilm, we quantified the proportion of dead cells in both WT and ΔRM biofilms using confocal microscopy with live/dead staining. Our results indicated no significant difference in the proportion of dead cells between the WT and ΔRM biofilms, suggesting that the observed increase in eDNA is not attributable to cell lysis (Fig. S2 in the supplemental material). In both gram-negative and gram-positive bacteria, MVs have long been identified as a source of eDNA (41). To explore this possibility, we isolated MVs from both WT and ΔRM biofilms and analyzed them using nanoparticle tracking analysis (NTA). The average diameter of MVs from WT and ΔRM biofilms was similar between strains—103.87 ± 7.45 nm for WT and 104.37 ± 1.95 nm for ΔRM. However, the ΔRM biofilm produced approximately twice as many MVs as the WT biofilm, with concentrations of 9.2 ± 0.4 (×10¹¹) particles/mL for WT and 15.8 ± 1.0 (×10¹¹) particles/mL for ΔRM (*P* = 0.0005; Fig. 4A). TEM analysis with uranyl acetate staining confirmed the characteristic “cup-shaped” morphology of MVs in both strains, although their observed size (20–100 nm) was smaller than that measured by NTA (Fig. 4B). Further quantification of DNA using SYTO-9 revealed that the elevated levels of eDNA in ΔRM biofilms correlated with the increased abundance of MVs (Fig. 4C), supporting the hypothesis that MVs contribute to eDNA accumulation in the absence of DAM.

### Transcriptome analysis of ΔRM mutant biofilm

**Fig 5:**
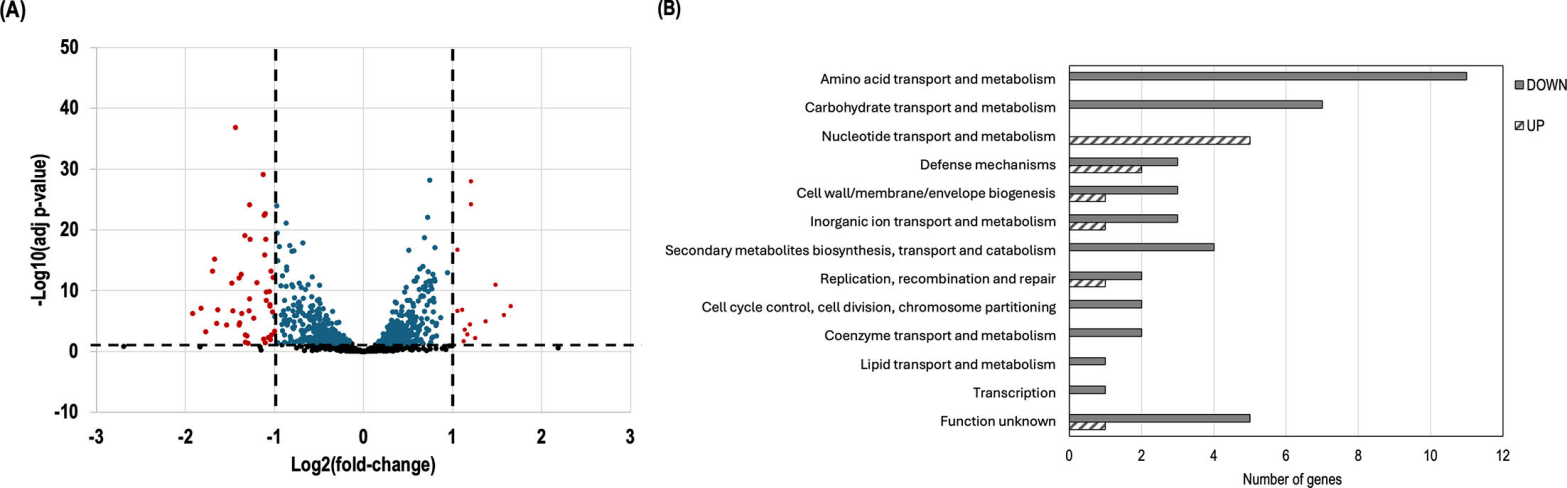
Overview of RNA-seq. (**A**) Volcano plot displaying differentially expressed genes (DEGs) between WT and ΔRM biofilms. Volcano plot shows the distribution of significance [−log_10_(adj *p*-value)] vs fold change [log_2_(fold-change)] for all genes. The vertical lines indicate a log_2_ (fold-change) cutoff ±1.0. The horizontal line represents the statistical significance threshold of adjusted *P*-value ≤ 0.05. Red dots: *p*-value and log_2_(fold-change); blue dots: *P*-value; black dots: nonsignificant. (**B**) COG functional of the identified proteins. A total of 11 upregulated and 44 downregulated genes were identified in the ΔRM mutant.

Given the significant alterations in the biofilm matrix composition observed in the ΔRM mutant, including increased eDNA levels and changes in MV production, we sought to understand the underlying molecular mechanisms driving these phenotypic changes. To achieve this, we performed RNA-seq analysis of the transcriptomes of WT and ΔRM mutant biofilm cells to identify global changes in gene expression associated with the absence of DAM. The volcano plot displayed the global transcriptional response of ΔRM biofilm cells (Fig. 5A). Compared to WT biofilm cells, a total of 60 DEGs (*P*-value ≤ 0.05 and log_2_FC ± 1.0) were identified in the ΔRM mutant. Of these DEGs, 46 were downregulated and 14 were upregulated (Table S1 in the supplemental material). The differential expression of randomly selected genes identified by RNA-seq was validated using RT-qPCR. The RT-qPCR results were consistent with the expression trends observed in the RNA-seq (Table S2 in the supplemental material). A cluster of orthologous gene (COG) distribution analysis was performed using the MicroScope platform. A total of 55 DEGs showing significant homology to those in the COG database were functionally grouped into 13 categories (Fig. 5B).

The most significant biological processes differentially regulated in the ΔRM mutant biofilm cells included amino acid transport and metabolism. Specifically, 11 genes were downregulated, primarily involved in histidine biosynthesis (*hisI*, *hisF*, *hisA*, *hisH*, *hisB*, *serB*, and *hisD*). Carbohydrate transport and metabolism also exhibited changes, with five genes (*msmF*, *msmG*, *gtfA*, *msmK*, and *dexB*) related to the multiple sugar-binding transport system being downregulated. Additionally, two genes (*glgD* and *glgB*) involved in the glycogen biosynthesis pathway were repressed. Furthermore, nucleotide transport and metabolism showed upregulation of five genes (*purC*, *purL*, *purF*, *purM*, and *purN*), contributing to *de novo* biosynthesis of purine nucleotides. Interestingly, we did not find significant downregulation of the purine operon repressor, *purR*, suggesting that another regulatory mechanism may be involved here. However, we note that PurR activity can also be modulated post-transcriptionally by purine metabolites, and thus its regulatory function may still be affected in the ΔRM mutant.

We also observed significant changes in cell wall/membrane biogenesis, which is particularly interesting due to its possible impact on biofilm phenotypes. In this category, four genes were identified, with one gene upregulated and three genes downregulated (Table 1). Interestingly, the gene *gtfC*, encoding a key virulence factor of *S. mutans*, was repressed in the ΔRM mutant biofilm. GtfC is crucial for synthesizing glucans that help *S. mutans* adhere to tooth enamel, supporting biofilm formation. Its reduced expression suggests a potential reduction in the structural integrity of the biofilm. Another noteworthy result is the upregulation of the gene SMU.2147c, which encodes a putative cell surface protein with homology to the aggregation-promoting factor in *Lactobacillus*. This finding is particularly interesting because, in our previous study published earlier this year, we demonstrated that the absence of DAM conferred an autoaggregative phenotype in planktonic cells (35). The upregulation of SMU.2147c may therefore be related to changes in biofilm characteristics, contributing to the observed fragility of the ΔRM mutant biofilm.

**TABLE 1:** Cell wall/membrane biogenesis-related genes

Gene	Functional protein	log_2_FC ΔRM vs WT
SMU.1005	Glucosyltransferase GtfC	−1.67
SMU.575c	CidA/LrgA	−1.04
SMU.836	LytF-like peptidoglycan hydrolase	−1.00
SMU.2147c	Putative cell surface protein	+1.21

### DAM-dependent transcription of the *purC* gene

**Fig 6:**
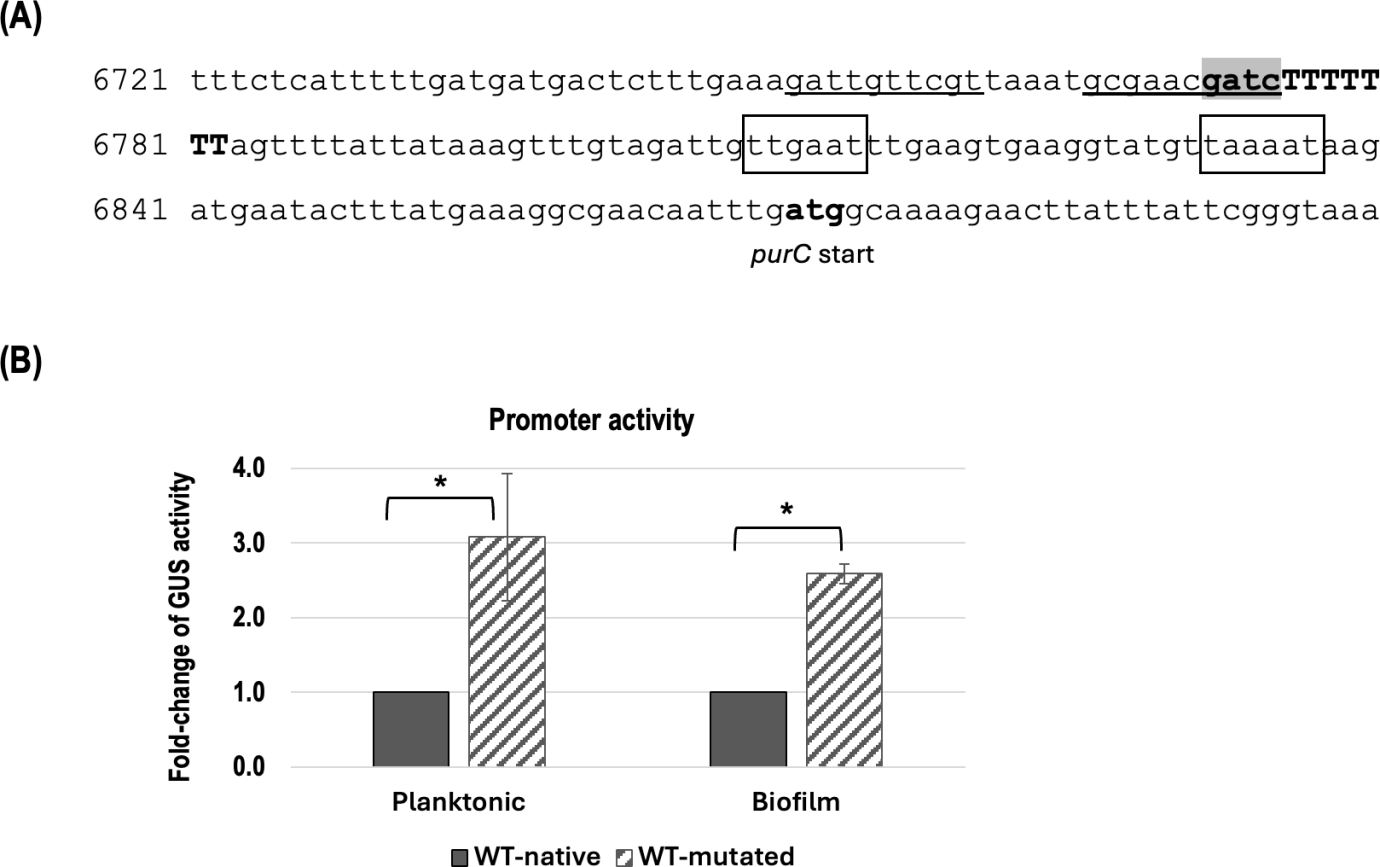
Promoter activity. (**A**) Nucleotide sequence of the promoter region of the *purC* gene in *S. mutans* UA159. The start codon (ATG) is shown in bold. Putative promoter sites of *purC* (−35, -10) are boxed. The predicted stem-loop terminator is underlined, and the T-rich region is shown in uppercase. The methylation site, GATC, is shown in a shaded region. (**B**) Fold change in promoter activity. Cells were harvested at mid-log phase (planktonic) and from 6 h biofilms. The *gusA* gene encoding a ß-glucuronidase enzyme was used as a reporter gene to measure the activity of the native promoter (GATC) and its mutated form (GGTC). The data are presented as fold-change in promoter activity, with the GUS activity of the native promoter set to 1.0. * denotes a statistically significant difference compared to WT (*P* < 0.05) under the same conditions (*N* = 3).

Although transcriptomic data provided evidence for the regulation of gene expression by DNA methylation, we aimed to confirm DAM-dependent transcriptional control through mutational analysis of putative DAM targets. We first screened for GATC sites located in promoter regions within 200 bp upstream of the start codon of genes identified by RNA-seq. Bioinformatic analysis identified a 5′-GATC-3′ site located 34 nucleotides upstream of the predicted −35 promoter box of the *purC* gene (Fig. 6A), which encodes SAICAR (phosphoribosylaminoimidazolesuccinocarboxide) synthase, a key enzyme in purine biosynthesis. Notably, this GATC site is situated within a predicted Rho-independent terminator, suggesting a potential regulatory role. To assess the functional significance of this methylation site, we mutated the GATC sequence within the *purC* promoter by substituting adenine (A) with guanine (G) at the second position, generating a non-methylatable GGTC variant. The “native” (GATC) and “mutated” (GGTC) promoter sequences were cloned upstream of the β-glucuronidase (*gusA*) reporter gene in pIB107 and integrated into the WT strain. The constructs did not affect *S. mutans* growth under the conditions tested (data not shown). As expected, the mutated promoter exhibited approximately a threefold increase in GUS reporter activity compared to the native, methylatable promoter (Fig. 6B). This result supports the hypothesis that adenine methylation at the GATC sites represses *purC* transcription and that disruption of this site relieves repression. The increase in promoter activity was consistently observed in both biofilm and planktonic cells. These findings highlight the significant role of DAM in regulating gene expression in *S. mutans*.

## DISCUSSION

Biofilm formation in *S. mutans* is a multifaceted process that plays a crucial role in the pathogenesis of dental caries (23, 26, 29). While the genetic factors involved in biofilm development have been extensively studied, the role of epigenetic modifications, such as DNA methylation, remains less explored. Our research aims to fill this gap by exploring the impact of adenine methylation on biofilm formation in *S. mutans*. In previous work, we discovered that the absence of adenine methylation—where adenine is the only methylated base in *S. mutans*—led to an autoaggregative phenotype in liquid cultures, which was dependent on eDNA (35). This finding prompted us to investigate further, given that aggregation is a key early step in biofilm development (42). In this study, we found that the loss of DAM resulted in notable changes in biofilm characteristics, including decreased exopolysaccharides and a puzzling increase in eDNA within the biofilm matrix. This increase in eDNA was linked to a higher production of MVs in the ΔRM mutant biofilms. We propose that the enhanced eDNA-dependent autoaggregation observed in planktonic cultures may contribute to the fragile biofilm phenotype of the ΔRM mutant. While eDNA promotes initial cell-to-cell interactions and surface attachment, excessive accumulation of eDNA in the absence of sufficient exopolysaccharide production may compromise the structural cohesion of the biofilm. This imbalance could weaken cell-to-cell and cell-matrix interactions, promote dispersal, and result in a loosely organized biofilm that is more susceptible to physical disruption (41, 43).

Initially, it was believed that the thick cell wall of gram-positive bacteria would prevent MV formation (20). However, research since the 1990 s has demonstrated that this is not the case. In gram-positive bacteria, MVs are formed when a portion of the cytoplasmic membrane protrudes and buds off, encapsulating various components before passing through the cell wall (44). The first report of MVs in *S. mutans* revealed that these MVs carry eDNA and proteins that facilitate biofilm formation, including the multifunctional adhesin P1, glucosyltransferase B (GtfB), and glucan-binding protein C (GbpC) (45). *S. mutans* MVs not only promote the formation of their own biofilms but also contribute to the development of *Candida albicans* biofilms, a common fungal species in the oral cavity often with early childhood caries (46). These findings underscore the importance of MVs in microbial interactions and biofilm dynamics. Therefore, the increased MV production observed in the ΔRM mutant was unexpected. Although the mechanisms of vesiculogenesis remain incompletely understood, MV biogenesis is known to be a highly regulated and active process (17).

Although our transcriptomic analysis was not initially designed to investigate MV production, it provided valuable insights into potential regulatory pathways that may influence this phenotype. Notably, we observed downregulation of several genes within the mutanobactin biosynthetic locus, including *mubP*, a homolog of the *sfp* gene in *Bacillus subtilis*, which is involved in the production of the lipoprotein surfactin (47). In *B. subtilis*, inactivation of *sfp* leads to a biofilm-defective phenotype despite increased MV accumulation (48). Similarly, previous work has shown that inactivation of mutanobactin in *S. mutans* results in increased MV production (49), suggesting that mutanobactin may promote MV turnover or lysis. Mutanobactin is a nonribosomal lipoprotein known to interact with and disrupt cellular membranes (40), and its downregulation in the ΔRM mutant may reduce MV disruption, thereby contributing to the observed increase in MV abundance. Consistent with this, our mutanobactin-deficient mutant exhibited elevated eDNA levels compared to the WT, though not to the same extent as the ΔRM mutant (Fig. S3). These findings support the idea that mutanobactin contributes to MV dynamics, likely by facilitating MV disruption. Interestingly, our RNA-seq data did not show differential expression of genes encoding di-adenyl cyclase or cyclic-di-AMP phosphodiesterase—enzymes known to regulate MV biogenesis via cyclic-di-AMP signaling (49). This observation reinforces the hypothesis that the elevated MV production in the ΔRM mutant is more closely linked to reduced expression of mutanobactin-related genes than to changes in cyclic-di-AMP metabolism. Additionally, the increase in MV production may represent a compensatory response to the reduced exopolysaccharide synthesis observed in the ΔRM mutant. MVs are known to support biofilm formation by mediating intercellular communication, transporting bioactive molecules, and contributing to the structural integrity of the biofilm (17). Thus, enhanced MV production could help maintain biofilm architecture in the context of diminished exopolysaccharide output. To further elucidate the molecular mechanisms involved, a comparative proteomic analysis of MVs from WT and ΔRM strains would be valuable to identify additional factors contributing to the altered biofilm phenotype.

Surprisingly, only few genes traditionally associated with biofilm formation were differentially expressed, suggesting that the biofilm-defective phenotype may not be solely driven by canonical biofilm-related pathways. However, several key genes with well-established roles in biofilm matrix development were significantly affected. In particular, the downregulation of *gtfB* and *gtfC*, which encode glucosyltransferases essential for the synthesis of water-insoluble glucans, likely contributes to the reduced structural integrity of the mutant biofilm. Additional changes, including the downregulation of *glgB* (a glucan branching enzyme) and *dexB* (a glucosidase), further support a shift in the composition and architecture of the extracellular matrix. These alterations may collectively weaken the cohesion and stability of the biofilm. Thus, while the number of differentially expressed biofilm-related genes was relatively small, their functional significance is likely substantial. In addition to these direct effects, transcriptomic data suggest that the ΔRM mutant undergoes broader metabolic reprogramming that may indirectly influence biofilm architecture. One notable example is the upregulation of genes involved in *de novo* purine nucleotide biosynthesis, including *purC*, *purL*, *purF*, *purM*, and *purN*. Notably, the DAM target site identified upstream of *purC* lies within a predicted rho-independent terminator region, which consists of a stem-loop structure followed by a T-rich sequence. The GATC motif located within the second stem of this structure, and its substitution to GGTC, not only prevents methylation but may also weaken the stability of the stem-loop, potentially affecting transcription termination. This structural impact could contribute to the observed increase in *purC* expression. In *Staphylococcus aureus*, purine metabolism has been shown to play a critical role in biofilm formation (50). A transcriptomic analysis revealed that *de novo* purine biosynthesis pathway was consistently upregulated under biofilm-inducing conditions, and that disruption of this pathway—either genetically or pharmacologically—significantly impairs biofilm formation (50). These findings suggest that purine biosynthesis supports the increased nucleotide demand associated with biofilm establishment. A similar link has been observed in *E. coli*, where purine metabolism influences biofilm formation through its impact on nucleotide pools and cyclic-di-GMP signaling (51). In our study, the elevated purine nucleotide biosynthesis in the ΔRM mutant may therefore be associated with the increased levels of eDNA observed in its biofilms. This increase in eDNA could represent an adaptive response aimed at maintaining biofilm integrity in the face of altered matrix composition. We also observed a modest downregulation (1.5-fold) of *purR*, the purine operon repressor. A previous study reported that deletion of *purR* in *S. aureus* led to increased eDNA release, suggesting that derepression of purine biosynthesis may contribute to this phenotype (52). Although the ΔRM mutant does not directly target *purR*, its reduced expression may result in partial derepression of the purine pathway, thereby contributing to the observed transcriptional changes and eDNA accumulation. While causality remains to be fully established, these findings support a potential regulatory link between purine metabolism and biofilm matrix composition. Additionally, we observed significant downregulation of genes involved in histidine biosynthesis. Given the known metabolic association between purine and histidine biosynthesis via the aminoimidazole carboxamide ribotide (AICAR) component of the purine-histidine-thiamine metabolic network (53), and emerging evidence in gram-positive bacteria suggesting a role for histidine metabolism in biofilm formation (54), these findings may reflect broader metabolic shifts contributing to the altered biofilm phenotype.

The genome of *S. mutans* UA159 contains 4,840 GATC methylation sites, yet our RNA-seq analysis identified only a limited number of differentially expressed genes in the ΔRM mutant biofilm. This is consistent with previous observations that, although adenine methyltransferases modify numerous sites across bacterial genomes, most of these modifications have minimal or no impact on gene regulation (8). However, a select subset of methylation events can exert significant regulatory effects, influencing the expression of key genes. This suggests that epigenetic regulation may act in a highly targeted manner, conferring selective or phenotypic advantages under specific conditions. Although only a few genes appeared to be affected in this study, we uncovered a novel and unexpected link between purine metabolism and biofilm formation in *S. mutans*—a connection that was not anticipated at the onset. This finding illustrates how epigenetic changes can reveal previously unrecognized regulatory mechanisms. It also opens the door to the possibility that additional genes or systems influenced by methylation remain to be discovered.

In conclusion, our study underscores the significant role of DNA methylation in shaping the biofilm phenotype of *S. mutans*. The absence of DAM resulted in notable changes in biofilm characteristics, including MV production and elevated eDNA levels. These findings highlight the importance of MVs in biofilm dynamics and suggest that the observed biofilm defect may be driven by the reduced expression of mutanobactin-related genes and the upregulation of purine nucleotide biosynthesis genes. The unexpected connection between purine metabolism and biofilm formation exemplifies how epigenetic regulation can uncover novel pathways and mechanisms, offering new perspectives on microbial adaptation and potential therapeutic targets. Importantly, since adenine methylation is rare or absent in mammalian cells, targeting adenine methyltransferase offers a promising and selective antimicrobial strategy with minimal risk of host toxicity.

## MATERIALS AND METHODS

### Strains and culture conditions

All strains used in this study are listed in Table S3 in the supplemental material. *S. mutans* strains were grown in Brain Heart Infusion (BHI) broth and incubated statically at 37°C in air with 5% CO_2_. When needed, cultures were supplemented with chloramphenicol (10 µg/mL), erythromycin (10 µg/mL), kanamycin (300 µg/mL), and/or spectinomycin (1 mg/mL). *E. coli* DH10B cells were grown in Luria-Bertani (LB) medium with aeration at 37°C. *E. coli* strains carrying plasmids were grown in shaken cultures of LB broth containing chloramphenicol (20 µg/mL) or kanamycin (50 µg/mL).

### Mutant strain construction and DNA manipulation

All plasmids used in this study are listed in Table S3, and the oligonucleotide sequences are listed in Table S4 (supplemental material). DNA (both circular and linear) was introduced into *S. mutans* UA159 or UA140 strains and their mutants by natural transformation (55). For the generation of nonpolar insertion-deletion mutants, allelic replacement mutations were performed by PCR ligation mutagenesis using antibiotic resistance cassettes (erythromycin or spectinomycin), as previously described (56). For ectopic gene expression, the full-length coding regions of the genes of interest were PCR-amplified using UA159 genomic DNA as the template. The PCR products were double-digested with BamHI/XhoI and cloned under the control of the constitutive lactococcal promoter P23 into the pIB166 vector. For the construction of the reporter strain, the predicted promoter region located upstream of the target gene was PCR-amplified from UA159 genomic DNA, double-digested with BamHI/XhoI, and cloned upstream of the *gusA* gene encoding *b*-glucuronidase enzyme into the promoterless vector pIB107. For site-directed mutagenesis, the sequence 5′-GATC-3′ was substituted with 5′-GGTC-3′ using the QuikChange XL Site-Directed Mutagenesis Kit (Agilent Technologies) following the manufacturer’s recommendations. Plasmid pHZ5 served as the template, and mutagenic primers CMT-1787 and CMT-1788 were used to introduce the A-to-G substitution. The resulting plasmid was designated as pHZ6 and confirmed by DNA sequencing.

### Biofilm assays

Static 24-h-old biofilms were developed in microtiter plates containing BHI broth supplemented with 1% (wt/vol) sucrose. The medium was refreshed after the first 8 h of incubation. For staining, 0.02% (wt/vol) crystal violet was added to each well and incubated at room temperature for 15 min before the solution was aspirated. For optical coherence tomography (OCT) imaging, the VivoSight Multi-Beam Swept Source OCT system (Michelson Diagnostics Ltd, UK) was used to obtain cross-sectional images of biofilms cultivated in BHI-sucrose on saliva-coated hydroxyapatite (sHA) disks (9.5 mm × 1.8 mm, Clarkson Chromatography Products). This system employs a class I laser (λ = 1305 nm) and scans at a rate of 10 kHz (57). The default scanning volume was set to 6 × 6 mm with an approximate penetration depth of 2 mm. For each sample, a total of 120 frames were recorded over this volume, with a pixel size of 4.65 µm. Prior to imaging, biofilms on HA disks were submerged in phosphate-buffered saline (PBS, pH 7.2).

### Quantification of biofilm exopolysaccharides

Exopolysaccharides were quantified using laser scanning confocal fluorescence microscopy as previously described (58, 59). Briefly, 1 µM Texas-Red-labeled dextran (Dextran, Texas Red, 70,000 MW, Neutral, 595/615 nm, Invitrogen) was added to the culture medium throughout the development of the 24-h-old biofilm. All bacterial cells in the biofilms were labeled with 5 µM SYTO 9 green-fluorescent nucleic acid stain (485/498 nm, Invitrogen) using standard protocols (58). For live/dead staining, 1.25 µM SYTO 9 and 2.5 µM propidium iodide were incubated with 24-h-old biofilms for 15 min prior to imaging. Images were acquired using a Zeiss LSM800 confocal microscope equipped with a 40 × oil immersion objective lens (CAMiLoD, University of Toronto). Ten Z-stack images were collected by biofilm at 0.23 µm intervals (512 by 512 pixels for quantification or 1,024 by 1,024 pixels for visualization in tagged image file format). Three-dimensional (3D) reconstructions of the biofilms were generated using Imaris 10 software (Bitplane, Switzerland). The biovolume of exopolysaccharides was calculated using Comstat2 software (*N* = 10) (60).

### Quantification of biofilm matrix proteins

The biofilm matrix was extracted using EDTA as previously described (61). Briefly, 24-h-old biofilms were developed in six-well polystyrene microtiter plates using BHI-sucrose. Biofilm samples from each well were resuspended in 1 mL of 0.9% (wt/vol) NaCl containing 2% (wt/vol) EDTA and incubated for 1 h at 4°C. The samples were then transferred to microcentrifuge tubes and centrifuged to collect the biofilm material. The resulting pellet was resuspended in 1 mL of a 1:1 solution of 0.9% (wt/vol) NaCl and 2% (wt/vol) EDTA, followed by an additional 1 h incubation at 4°C. The supernatant was harvested by centrifugation at 10,000 × *g* for 30 min at 4°C and filter-sterilized using a 0.45 µm pore size hydrophilic PVDF membrane. Protein concentrations were determined using the Bradford assay according to the manufacturer’s instructions.

### Quantification of biofilm eDNA

One-day-old biofilms were developed in six-well polystyrene microtiter plates as described above. Biofilm samples were washed twice with 0.9% (wt/vol) NaCl, harvested in 1 mL of 0.9% (wt/vol) NaCl, and transferred into microcentrifuge tubes. To disrupt the biofilms and isolate eDNA from the matrix, the suspensions were sonicated on ice (10 s, twice at 20% amplitude with 1 min intervals) using a sonicator (XL-2000, Qsonica, USA). The suspensions were then centrifuged at 10,000 × *g* for 10 min at 4°C. The supernatant was collected and filtered through 0.22 µm syringe filters (Millipore, USA). SYTO-9 green (Invitrogen) was added to the supernatants to quantify eDNA using a fluorescence microplate reader (HIDEX) with excitation at 485 nm and emission at 525 nm.

### Scanning electron microscopy

Biofilms (6 h and 24 h) were developed on plastic coverslips (13 mm) in BHI-sucrose. After incubation, biofilms were gently washed with PBS (pH 7.2) and fixed in 1 mL of 3.7% (vol/vol) formaldehyde in PBS for 24 h at room temperature. The samples were then dehydrated through a series of ethanol solutions (30%, 50%, 70%, 95%, and 100% (vol/vol)). Finally, the dehydrated samples were rinsed twice with hexamethyldisilane, treated with gold spray, and examined under a scanning electron microscope (Hitachi FlexSEM 1000; CAMiLoD, University of Toronto).

### Cell surface characterization

Zeta potential was measured using *S. mutans* cells resuspended in 10 mmol/L KCl solution and adjusted to an optical density of approximately 0.05 at 600 nm (OD₆₀₀). Measurements were performed using phase analysis light scattering (PALS) with a NanoBrook Omni instrument (Brookhaven Instruments). Cell surface hydrophobicity was assessed using the microbial adhesion to hydrocarbon (MATH) assay. Briefly, *S. mutans* cells were washed twice with PBS (pH 7.2) and resuspended to an OD₅₅₀ of approximately 0.4 (OD₁). A 2 mL aliquot of this bacterial suspension was mixed with varying volumes of toluene (0.1, 0.2, 0.3, or 0.4 mL) in glass tubes, vortexed for 5 min, and allowed to stand at room temperature for 10 min. The absorbance of the aqueous phase was then measured at OD₅₅₀ (OD₂). Cell surface hydrophobicity was calculated using the following equation: Relative turbidity (%) = OD_2_/OD_1_ × 100.

### Membrane vesicle (MV) preparation and analysis

One-day-old biofilms, developed as described above, were harvested in PBS, vortexed for 5 min, and collected by centrifugation. The supernatant was filtered through 0.22 µm syringe filters (Millipore, USA) and transferred into ultracentrifuge tubes. Samples were subjected to ultracentrifugation at 120,000 × *g* for 2 h at 4°C using a SW 28 rotor (Optima L-70, Beckman Coulter, Germany). The supernatant was carefully discarded, and the resulting pellet was resuspended in 500 µL of filtered PBS. After vortexing for 20 s, the suspension was syringe-filtered again using a 0.22 µm filter prior to further analysis. The concentration and size distribution of MVs were determined using nanoparticle tracking analysis (NTA; NanoSight 300, Malvern) at the Structural & Biophysical Core Facility, The Hospital for Sick Children, Toronto, Canada. All samples were diluted 1:500 in filtered PBS to ensure vesicle concentrations remained within the optimal range of 20–200 particles per frame. The diluted samples were introduced into a chamber illuminated by a green laser, and high-sensitivity video recordings (Camera level 14) were captured. Three 30 second videos were recorded and analyzed using NanoSight 3.1 software. MV-associated DNA levels were quantified using SYTO-9, as described above. For transmission electron microscopy (TEM), vesicle samples were fixed in a 1:1 ratio with 4% (wt/vol) paraformaldehyde and incubated at 4°C for at least 24 h. Samples were then stained with 2% (wt/vol) uranyl acetate, and images were acquired using a Hitachi HT7800 TEM operated at 120 kV (Nanoscale Biomedical Imaging Facility, The Hospital for Sick Children, Toronto, Canada).

### RNA-Sequencing (RNA-Seq) and data analysis

Static 6-h-old biofilms were developed in microtiter plates containing BHI-sucrose. This time point was selected to capture the early stages of biofilm development in *S. mutans*, including initial attachment and matrix production, prior to biofilm maturation (62). Total RNA was extracted from biofilm samples of *S. mutans* UA159 WT and its ΔRM mutant using the RiboPure RNA Purification Kit (Fisher). All experimental groups were performed in triplicate. RNA concentrations were measured using a NanoDrop spectrophotometer, and RNA quality was assessed with an Agilent 2100 Bioanalyzer. The cDNA libraries were prepared using the NGS Illumina Stranded Total RNA Prep Ligation kit with Ribo-Zero Plus, following the manufacturer’s protocol. Sequencing was performed on the Illumina MiSeq platform using a Flowcell at the Center for Applied Genomics (The Hospital for Sick Children, Toronto, Canada). Data analysis was conducted as described in our previous studies (34, 35). Sequencing reads were mapped to the *S. mutans* UA159 reference genome (NC_004350.2). Genes were considered differentially expressed when the Benjamini-Hochberg multiple adjusted *p*-value was below 0.05. Significantly differentially expressed protein-coding genes were classified into clusters of orthologous groups (COGs) using the MicroScope platform (v3.16.1).

### Promoter activity analysis

Reporter strains were harvested by centrifugation from overnight cultures or 6 h biofilms. Cells were lysed by sonication using an XL-2000 sonicator (Qsonica, USA) at power setting 8 for 1 min on ice, and intracellular β-glucuronidase (GUS) activity was measured as previously described (63). Briefly, equal volumes of intracellular fractions and GUS buffer (100 mM Na_2_HPO_4_, pH 7.0; 20 mM *β* mercaptoethanol; 2 mM EDTA; 0.2% (vol/vol) Triton X-100; and 1 mM *para*-nitrophenyl-*β*-D-glucuronide) were mixed and incubated at 37°C for 3 h. The reaction was stopped by adding 3.5 mM Na_2_CO_3_, and absorbance was measured at 420 nm (A_420_). GUS activity was calculated in Miller Units (MUs) using the formula: MUs = (1000 × A_420_) / (time [min] × OD_600_).

### Statistical analysis

All assays were carried out in triplicate, and data were analyzed using GraphPad Prism 9.0. For comparisons of two groups, an unpaired *t*-test was used. For multiple comparisons, one-way ANOVA was used, and a *P*-value of ≤0.05 was considered statistically significant.

## Data Availability

The data that support the findings of this study are openly available in Gene Expression Omnibus at https://www.ncbi.nlm.nih.gov/geo/, reference number GSE265916.

## SUPPLEMENTAL MATERIAL

Supplemental material aem.01094-25-s0001.pdf Tables S1 to S4; Fig. S1 to S3.

ASM does not own the copyrights to Supplemental Material that may be linked to, or accessed through, an article. The authors have granted ASM a non-exclusive, world-wide license to publish the Supplemental Material files. Please contact the corresponding author directly for reuse.
