# Supplementary material for: DNA adenine methylation influences gene expression and biofilm formation in Streptococcus mutans
Source: Appl Environ Microbiol. 2025 Sep 23;91(10):e01094-25. doi: 10.1128/aem.01094-25 (PMC12542736; doi:10.1128/aem.01094-25)
Supplement: Supplemental material — Tables S1 to S4; Fig. S1 to S3. [file aem.01094-25-s0001.pdf]

SUPPLEMENTAL MATERIAL

DNA adenine methylation influences gene expression and biofilm formation in *Streptococcus mutans*

Zhao *et al.*, 2025

TABLE S1. DEGs significantly (adj *p*-value ≤0.05) differentially regulated (log_2FC ±1.0) in *S. mutans* ΔRM mutant biofilm. *S. mutans* locus tag as in GenBank (UA159 accession no. AE014133).

Locus tag	Gene	Product	log_2FC
SMU_RS01225	SMU.239c	VanZ family protein	−1.92
SMU_RS05840	SMU.1267c	YrdB family protein	−1.82
SMU_RS05805	SMU.1260c	DivIVA domain-containing protein	−1.77
SMU_RS05040	SMU.1093	ABC transporter permease	−1.70
SMU_RS04625	SMU.1005	Glucosyltransferase GtfC	−1.67
SMU_RS05810	NA	Phosphoribosyl-ATP diphosphatase	−1.65
SMU_RS05815	SMU.1262c	Hypothetical protein	−1.64
SMU_RS07120	SMU.1570	Sugar ABC transporter permease	−1.54
SMU_RS05835	SMU.1266	Imidazole glycerol phosphate synthase subunit HisH	−1.48
SMU_RS05820	SMU.1263	Phosphoribosyl-AMP cyclohydrolase	−1.47
SMU_RS06985	SMU.1538	Glucose-1-phosphate adenylyltransferase	−1.44
SMU_RS01465	SMU.295	Hypothetical protein	−1.40
SMU_RS05825	SMU.1264	Imidazole glycerol phosphate synthase subunit HisF	−1.40
SMU_RS04055	SMU.874	Bifunctional homocysteine S-methyltransferase/methylenetetrahydrofolate reductase	−1.39
SMU_RS05830	SMU.1265	1-(5-phosphoribosyl)-5-[(5-phosphoribosylamino)methylideneamino]imidazole-4-carboxamide isomerase	−1.37
SMU_RS07125	SMU.1571	ABC transporter ATP-binding protein	−1.37
SMU_RS06150	SMU.1338c	Mutanobactin A system MFS transporter MubZ	−1.33
SMU_RS10070	NA	Hypothetical protein	−1.33
SMU_RS05045	SMU.1094	ABC transporter ATP-binding protein	−1.32
SMU_RS01045	SMU.199c	Conjugal transfer protein	−1.31
SMU_RS06390	SMU.1403c	CRISPR-associated endonuclease Cas2	−1.30
SMU_RS05845	SMU.1268	Imidazoleglycerol-phosphate dehydratase HisB	−1.29
SMU_RS05850	SMU.1269	Phosphoserine phosphatase SerB	−1.28
SMU_RS06155	SMU.1339	Mutanobactin A non-ribosomal peptide synthetase MubD	−1.28
SMU_RS05855	SMU.1270	Histidinol dehydrogenase	−1.27
SMU_RS07115	SMU.1569	Carbohydrate ABC transporter permease	−1.23
SMU_RS06980	SMU.1537	Glucose-1-phosphate adenylyltransferase subunit GlgD	−1.20
SMU_RS06990	SMU.1539	1,4-alpha-glucan branching protein GlgB	−1.13
SMU_RS05795	SMU.1258c	Restriction endonuclease	−1.12
SMU_RS06160	SMU.1340	Mutanobactin A non-ribosomal peptide synthetase MubC	−1.12
SMU_RS03890	SMU.839	Folylpolyglutamate synthase/dihydrofolate synthase family protein"	−1.11
SMU_RS04095	SMU.881	Sucrose phosphorylase GtfA	−1.11
SMU_RS01020	SMU.194c	ASCH/PUA domain-containing protein	−1.10
SMU_RS06140	SMU.1336	Mutanobactin A biosynthesis transacylase MubI	−1.10
SMU_RS04090	SMU.880	Carbohydrate ABC transporter permease	−1.09
SMU_RS04100	SMU.882	ABC transporter ATP-binding protein	−1.09

SMU_RS04050	SMU.873	5-methyltetrahydropteroyltriglutamate-homocysteine S-methyltransferase	−1.07
SMU_RS04760	SMU.1034c	Tyrosine recombinase XerS	−1.06
SMU_RS04085	SMU.879	Carbohydrate ABC transporter permease	−1.05
SMU_RS06135	SMU.1335c	Mutanobactin A biosynthesis reductase MubJ	−1.05
SMU_RS02575	SMU.536	Phosphoribosylanthranilate isomerase	−1.04
SMU_RS02750	SMU.575c	Holin-like protein LrgA	−1.04
SMU_RS06145	SMU.1337c	Mutanobactin A biosynthesis alpha/beta hydrolase MubM	−1.04
SMU_RS02455	SMU.510	Hypothetical protein	−1.02
SMU_RS04105	SMU.883	Glucan 1,6-alpha-glucosidase DexB	−1.02
SMU_RS03875	SMU.836	GBS Bsp-like repeat-containing protein	−1.00
SMU_RS08840	SMU.1945	Polyphosphate polymerase domain-containing protein	+1.05
SMU_RS00255	SMU.35	Phosphoribosylglycinamide formyltransferase PurN	+1.06
SMU_RS00250	SMU.34	Phosphoribosylformylglycinamidine cyclo-ligase PurM	+1.11
SMU_RS01720	NA	tRNA-Ser	+1.13
SMU_RS02435	NA	Hypothetical protein	+1.14
SMU_RS00280	SMU.40	Type II toxin-antitoxin system RelE/ParE family toxin	+1.17
SMU_RS00245	NA	Hypothetical protein	+1.20
SMU_RS08845	SMU.1946	DUF4956 domain-containing protein	+1.21
SMU_RS09795	SMU.2147c	LysM peptidoglycan-binding domain-containing protein	+1.21
SMU_RS00630	SMU.121	MATE family efflux transporter	+1.26
SMU_RS00225	SMU.29	Phosphoribosylaminoimidazolesuccinocarboxamide synthase PurC	+1.37
SMU_RS00240	SMU.32	Amidophosphoribosyltransferase PurF	+1.48
SMU_RS00230	SMU.30	Phosphoribosylformylglycinamidine synthase PurL	+1.58
SMU_RS00235	SMU.31	Putative DNA alkylation repair protein	+1.65

TABLE S2. Validation of RNA-Seq data by qRT-PCR. *S. mutans* locus tag as in GenBank (UA159 accession no. AE014133).

**Locus tag**	**Gene**	**Product**	**Fold-change ΔRM vs. WT (SD)**
SMU_RS00225	SMU.29	SAICAR synthase PurC	+3.46 (±0.76)
SMU_RS01485	SMU.299c	Putative bacteriocin peptide precursor Pep299	+1.70 (±1.92)
SMU_RS04080	SMU.878	Sugar-binding protein MsmE	+1.24 (±0.20)
SMU_RS04095	SMU.881	Sucrose phosphorylase GtfA	−2.97 (±1.06)
SMU_RS04105	SMU.883	Glucan 1,6-alpha-glucosidase DexB	−3.18 (±0.95)
SMU_RS04620	SMU.1004	Glucosyltransferase GtfB	−2.27 (±0.34)
SMU_RS04625	SMU.1005	Glucosyltransferase GtfC	−2.79 (±1.14)
SMU_RS06360	SMU.1396	Glucan-binding protein C GbpC	+1.38 (±1.10)
SMU_RS06390	SMU.1403c	CRISPR-associated endonuclease Cas2	−3.98 (±0.59)

TABLE S3. Bacterial strains and plasmids used in this study.

Strain or plasmid	Relevant characteristic(s)[a]	Source or reference
**Strains**		
S. mutans		
UA159 WT	Genome sequence reference strain	ATCC
ΔRM	*dpnMAB* mutant derived from UA159; Em^r or Sp^r	(1)
Δmub	*mub* gene cluster mutant derived from UA159, Em^r	This study
UA159::pHZ5	WT with pHZ5 integrated; Km^r	This study
UA159::pHZ6	WT with pHZ6 integrated; Km^r	This study
ΔRM::pHZ3(pHZ4)	ΔRM with pHZ3 integrated and harboring pHZ4; Em^r, Km^r, Cm^r	(1)
ΔRM::pHZ5	ΔRM with pHZ5 integrated; Em^r Km^r	This study
UA140	UA140 reference strain	ATCC
ΔRM-140	*dpnMAB*-like locus mutant derived from UA140; Em^r	(1)
ΔRM-140::pHZ3(pHZ4)	ΔRM-140 with pHZ3 integrated and harboring pHZ4; Em^r, Km^r, Cm^r	This study
E. coli		
DH10B	Host strain for cloning and plasmid production	Lab stock
**Plasmids**		
pIB107	Plasmid for chromosomal integration into *S. mutans*; Km^r	(2)
pIB166	Shuttle plasmid containing the P_{23} lactococcal promoter; Cm^r	(3)
pHZ3	P_{CON}-*dpnM* cloned into pIB107; Km^r	(1)
pHZ4	*dpnA* cloned under the control of P_{23} into pIB166; Cm^r	(1)
pHZ5	P_{PurC} transcriptionally fused to *gusA* into pIB107; Km^r	This study
pHZ6	Mutated P_{PurC} transcriptionally fused to *gusA* into pIB107; Km^r	This study

[a] Em^r, erythromycin resistance; Sp^r, spectinomycin resistance; Km^r, kanamycin resistance; Cm^r, chloramphenicol resistance.

References

(1) Zhao H, Dufour D, Zhong J, Gong SG, Roy PH, Lévesque CM. Decoding adenine DNA methylation effects in *Streptococcus mutans*: insights into self-DNA protection and autoaggregation. *Mol Oral Microbiol*. 2025;40(2):82−93. DOI:10.1111/omi.12489

(2) Biswas S, Biswas I. Regulation of the glucosyltransferase (*gtfBC*) operon by CovR in *Streptococcus mutans*. *J Bacteriol*. 2006;188(3):988−998.DOI:10.1128/JB.188.3.988-998.2006

(3) Biswas I, Jha JK, Fromm N. Shuttle expression plasmids for genetic studies in *Streptococcus mutans*. Microbiology, 2008, 154:2275−2282. DOI: 10.1099/mic.0.2008/019265-0

TABLE S4. Primers used in this study.

Primer	Gene	Sequence (5′ to 3′)[a]
**Promoter activity**		
CMT-1785	*purC*	F: 5′-GGATCCGGATCCTTGTTGAGATTGACTCTGGAG-3′
CMT-1786	*purC*	R: 5′-CTCGAGCTCGAGTCACATTTTCGTCTTCTGTCG-3′
**Mutagenesis**		
CMT-1788	*purC*	F: 5′-TTGAAAGATTGTTCGTTAAATGCGAACGGTCTTTTTTTAGTTTTATTATAAAGTTTGT-3′
CMT-1787	*purC*	R: 5′-ACAAACTTTATAATAAAACTAAAAAAAGACCGTTCGCATTTAACGAACAATCTTTCAA-3′
CMT-1797	*mub*	F: 5′-TGGATGCAAA ATGCGCATCG-3′
CMT-1798	*mub*	R: 5′-GGCGCGCCCAGCCAATAC CAGAATCTCC-3′
CMT-1799	*mub*	F: 5′-GGCCGGCCAGAAAGGATACAGCCTTTCTG-3′
CMT-1800	*mub*	R: 5′-CGAGTTGGTCAACTTTTCGG-3′
**RT-qPCR**		
CMT-220	16S rRNA	F: 5′-CTTACCAGGTCTTGACATCCCG-3′
CMT-221	16S rRNA	R: 5′-ACCCAACATCTCACGACACGAG-3′
CMT-1791	*purC*	F: 5′-AAGGTATTCAGTTGGAGAAGC -3′
CMT-1792	*purC*	R: 5′-GGCAATATCTTCATCGTTAGC -3′
CMT-1342	SMU.299	F: 5′-ACGATGGAGCTAATGGCTAT-3′
CMT-1343	SMU.299	R: 5′-AAGCGTAAGCGGCAAAACTT-3′
CMT-1721	SMU.878	F: 5′-ACACCGTTTGCTTTAGCGGG-3′
CMT-1722	SMU.878	R: 5′-ATTAGCGGCATTAGCACTGC-3′
CMT-1723	SMU.881	F: 5′-ACCCTTGATACACATGACGG-3′
CMT-1724	SMU.881	R: 5′-TGGCACCGACCTTATAAAGC-3′
CMT-1725	SMU.883	F: 5′-TCAATCTGGGGAAATACGGG-3′
CMT-1726	SMU.883	R: 5′-AAATATAAGGTGTCCCACGC-3′
CMT-1601	SMU.1004	F: 5′-TTACTGATGACATGGTGGCC-3′
CMT-1602	SMU.1004	R: 5′-GCTTGAATAGTTGCAGCTGC-3′
CMT-1654	SMU.1005	F: 5′-CCAAAATGGTATTATGGCTGTCG-3′
CMT-1655	SMU.1005	R: 5′-TGAGTCTCTATCAAAGTAACGCAG-3′
CMT-1737	SMU.1396	F: 5′-TGAACCAACGCCAGAAAAGC-3′
CMT-1738	SMU.1396	R: 5′-CACGCTCTCTAACACGCATTTC-3′
CMT-1727	SMU.1403	F: 5′-TTCAGTCACGGTCAACAAGG-3′
CMT-1728	SMU.1403	R: 5′-GCCAATACAGCCATGATTGC-3′

[a]F: Forward primer; R: Reverse primer.

[b]Underlined sequences denote the restriction sites used for cloning.

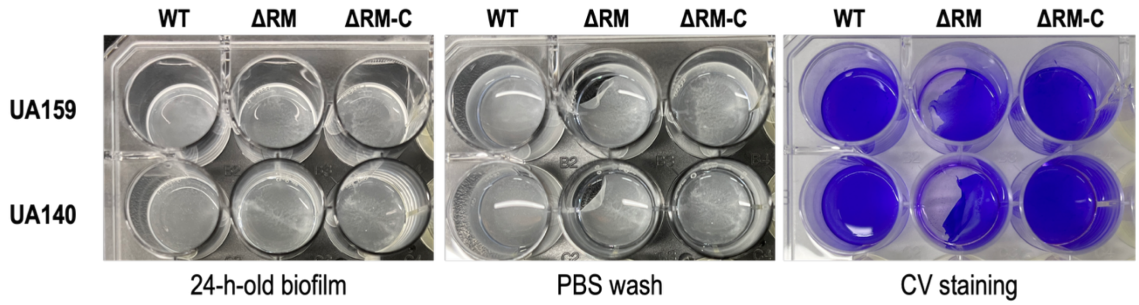

FIG S1. Biofilms of *S. mutans* UA159 and UA140 strains. Static biofilms (24-h-old) were developed in BHI-sucrose using microtiter plates. Biofilms were washed with phosphate-buffered saline (PBS, pH 7.2) and stained with crystal violet (CV). WT: wild-type strain; ΔRM: Dam deficient mutant; ΔRM-C: ΔRM mutant complemented with the *dpnM-dpnA* methyltransferase genes.

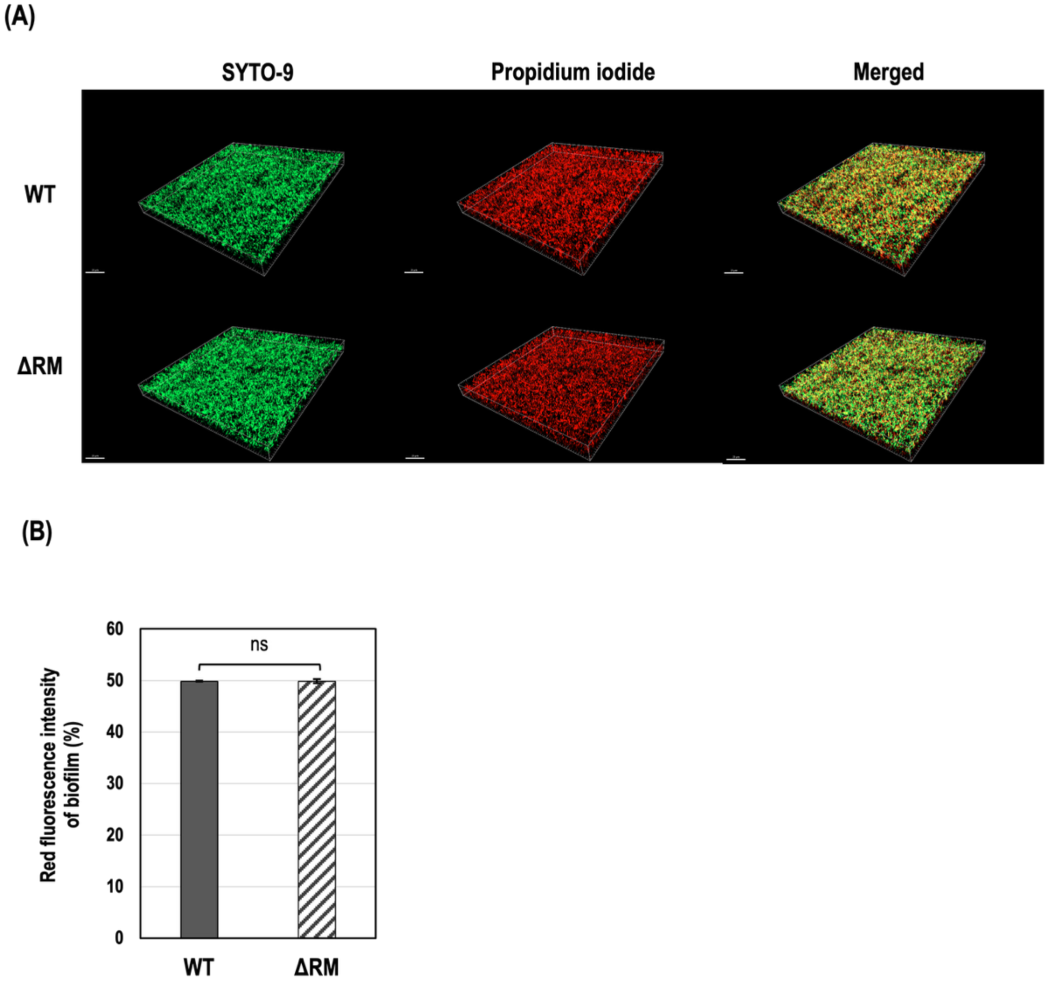

FIG S2. Live/Dead staining of *S. mutans* biofilms. Static biofilms (24-h-old) were developed in BHI-sucrose using microtiter plates. Biofilms were stained with SYTO-9 (green, 485/498 nm) for live bacterial cells and propidium iodide (red, 535/617 nm) for dead bacterial cells. **(A)** Representative CLSM 3D images of WT and ΔRM biofilms (magnification, ×40). **(B)** Percentage of red fluorescence intensity. ns: no significant difference (p >0.05; N=3).

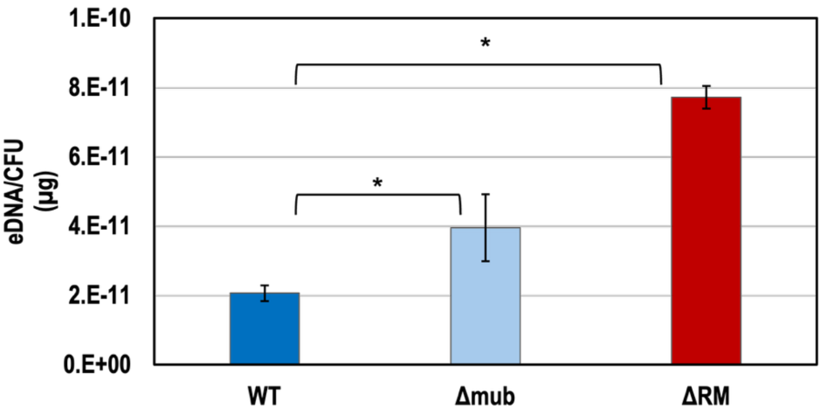

FIG S3. Quantification of eDNA in *S. mutans* biofilms. Static biofilms (24-h-old) were developed in BHI-sucrose using 6-well polystyrene microtiter plates. Biofilm samples were washed twice with 0.9% (w/v) NaCl, harvested in 1.0 mL of 0.9% (w/v) NaCl, and transferred into microcentrifuge tubes. To disrupt the biofilms and isolate eDNA from the matrix, the suspensions were sonicated on ice (10 s, twice at 20% amplitude with 1-min intervals) using a sonicator (XL-2000, Qsonica, USA). The suspensions were then centrifuged at 10,000 × *g* for 10 min at 4°C. The supernatant was collected and filtered through 0.22-µm syringe filters (Millipore, USA). SYTO-9 green (Invitrogen) was added to the supernatants to quantify eDNA using a fluorescence microplate reader (HIDEX) with excitation at 485 nm and emission at 525 nm. * denotes a statistically significant difference compared to WT (p <0.05; N=3).
